# Anatomical Variations of the Splenic Artery: Clinical and Surgical Implications—A Systematic Review

**DOI:** 10.3390/life16071077

**Published:** 2026-06-27

**Authors:** Cătălin Prodan-Bărbulescu, Laura Andreea Ghenciu, Carmen Neamțu, Rami Hajjar, Ionut Flaviu Faur, Ioana Adelina Faur, Cătălin Ionuț Vlăduț Feier, Norberth-Istvan Varga, Sorin Bolintineanu, Amadeus Dobrescu, Dan Brebu

**Affiliations:** 1 Department I—Discipline of Anatomy and Embryology, Faculty of Medicine, “Victor Babeş” University of Medicine and Pharmacy Timisoara, Eftimie Murgu Square 2, 300041 Timisoara, Romania; catalin.prodan-barbulescu@umft.ro (C.P.-B.); s.bolintineanu@umft.ro (S.B.); 2 2nd Surgery Clinic, “Pius Brinzeu” Clinical Emergency County Hospital, 300723 Timisoara, Romania; rami.hajjar@umft.ro (R.H.); flaviu.faur@umft.ro (I.F.F.); adelina.clim@umft.ro (I.A.F.); dobrescu.amadeus@umft.ro (A.D.); brebu.dan@umft.ro (D.B.); 3 Doctoral School, “Victor Babes” University of Medicine and Pharmacy Timisoara, Eftimie Murgu Square 2, 300041 Timisoara, Romania; norberth.varga@umft.ro; 4 Department III, Discipline of Pathophysiology, “Victor Babes” University of Medicine and Pharmacy, 300041 Timisoara, Romania; 5 Center for Translational Research and Systems Medicine, “Victor Babes” University of Medicine and Pharmacy, 300041 Timisoara, Romania; 6 Faculty of Pharmacy, “Victor Babes” University of Medicine and Pharmacy Timisoara, Eftimie Murgu Sq., Nr. 2, 30041 Timisoara, Romania; 7 Pathology Department, Clinical County Emergency Hospital of Arad, Andrenyi Karoly, Str., Nr. 2-4, 310037 Arad, Romania; 8 X Department of General Surgery, “Victor Babes” University of Medicine and Pharmacy, 300041 Timisoara, Romania; 9 First Discipline of Surgery, Department X-Surgery, “Victor Babes” University of Medicine and Pharmacy, E. Murgu Sq. 2, 300041 Timisoara, Romania; catalin.feier@umft.ro; 10 First Surgery Clinic, “Pius Brinzeu” Clinical Emergency Hospital, 300723 Timisoara, Romania; 11 Researching Future Chirurgie 2 (CHIR 2), Timisoara Emergency County Hospital, 300723 Timisoara, Romania

**Keywords:** splenic artery variations, vascular anatomy, microvasculature of the spleen, celiac trunk, accessory splenic artery, CT angiography of the splenic artery, splenic perfusion

## Abstract

Background: The splenic artery exhibits considerable anatomical variability in its origin, course, and branching pattern, with important implications for upper abdominal surgery. However, existing evidence remains fragmented across anatomical, radiological, and clinical studies. Objective: This systematic review aimed to synthesise current evidence on anatomical variations in the splenic artery and evaluate their surgical implications in upper abdominal procedures. Methods: A comprehensive literature search was conducted across PubMed, Scopus, Web of Science, and the Cochrane Library. Studies reporting anatomical variations in the splenic artery identified through cadaveric dissection, radiological imaging, or intraoperative findings were included. Data were extracted on origin, course, branching pattern, and accessory arteries, along with reported surgical implications. Methodological quality varied across study designs, with radiological and clinical studies generally demonstrating lower risk of bias than descriptive cadaveric investigations. Results: Seventeen studies involving 4301 subjects and specimens were included, encompassing cadaveric, radiological, and clinical investigations. The splenic artery most commonly originates from the celiac trunk, although less common origins from the abdominal aorta and the superior mesenteric artery have been reported. A suprapancreatic course was predominant, but retropancreatic and intrapancreatic variations were consistently documented. The artery frequently exhibited a tortuous configuration, and branching patterns were highly variable, with terminal bifurcation being most common. Segmental and polar arteries, as well as variations in gastric branches, were frequently observed. These vascular variations may influence operative complexity and increase the risk of intraoperative vascular injury, particularly during pancreatic, gastric, and splenic procedures. Conclusions: The splenic artery exhibits substantial anatomical variability with direct implications for surgical planning and outcomes. Preoperative vascular assessment using advanced imaging techniques may improve surgical safety in upper abdominal procedures.

## 1. Introduction

The splenic artery is a major branch of the celiac trunk and represents a clinically relevant vessel in upper abdominal surgery because of its variable origin, tortuous course, relationship with the pancreas, and diverse terminal branching patterns [1,2]. These anatomical features are particularly important during pancreatic, gastric, splenic, and endovascular procedures [2,3,4,5,6,7,8,9,10,11,12,13].

These variations may increase the risk of vascular injury, bleeding, ischemic complications, incomplete devascularization, or technical failure during distal pancreatectomy, spleen-preserving surgery, gastrectomy with lymph node dissection, splenectomy, and endovascular embolization. Therefore, accurate preoperative recognition of splenic artery variants is essential for surgical planning and procedural safety [5,10,14,15,16,17,18,19].

Thus, this systematic review aims to synthesise current evidence on anatomical variations in the splenic artery and to evaluate their clinical and surgical implications. Particular emphasis is placed on variations relevant to preoperative imaging assessment, surgical planning, and intraoperative safety.

## 2. Methodology—Literature Search Strategy

This systematic review was conducted and reported in accordance with the PRISMA 2020 guidelines. A comprehensive literature search was performed in PubMed, Scopus, Web of Science, and the Cochrane Library from database inception to March 2025. The PubMed search strategy combined terms related to the splenic artery and its variants, including “splenic artery”, “lienal artery”, “anatomical variation”, “vascular variation”, “branching pattern”, “course”, “origin”, “pancreatic surgery”, “pancreatectomy”, “gastrectomy”, “splenectomy”, “embolization”, and “computed tomography angiography”. Equivalent strategies were adapted for the other databases. Only studies involving human subjects or human cadaveric specimens and published in English were considered eligible.

Studies were included if they reported anatomical variations in the splenic artery identified through cadaveric dissection, radiological imaging, or intraoperative observations. Eligible study designs comprised cadaveric studies, radiological cohort studies, retrospective or prospective observational studies, and clinical case series. Case reports, narrative reviews, editorials, conference abstracts, animal studies, and non-English publications were excluded.

All records were screened by two independent reviewers using Rayyan software version 1.7.4. Titles and abstracts were first evaluated, followed by full-text assessment of studies potentially meeting the above-mentioned criteria. Disagreements were resolved by consensus or consultation with a third reviewer. Data were extracted using a standardised form that included study characteristics, sample size, methodological approach, splenic artery origin, course, branching pattern, accessory vessels, and reported clinical or surgical implications.

The methodological quality and risk of bias of the included studies were assessed independently by two reviewers using the Anatomical Quality Assurance tool. Disagreements were resolved by consensus. Inter-reviewer agreement was not formally quantified. Because of the heterogeneity of the included cadaveric, radiological, and clinical studies, a meta-analysis was not performed, and the GRADE approach was not formally applied. Data were synthesised narratively according to predefined anatomical domains: origin, course, branching pattern, accessory vessels, and surgical relevance.

The inclusion and exclusion criteria were defined according to the PECO framework and are summarized in Table 1. 

## 3. Results

### 3.1. Study Selection

**Figure 1:**
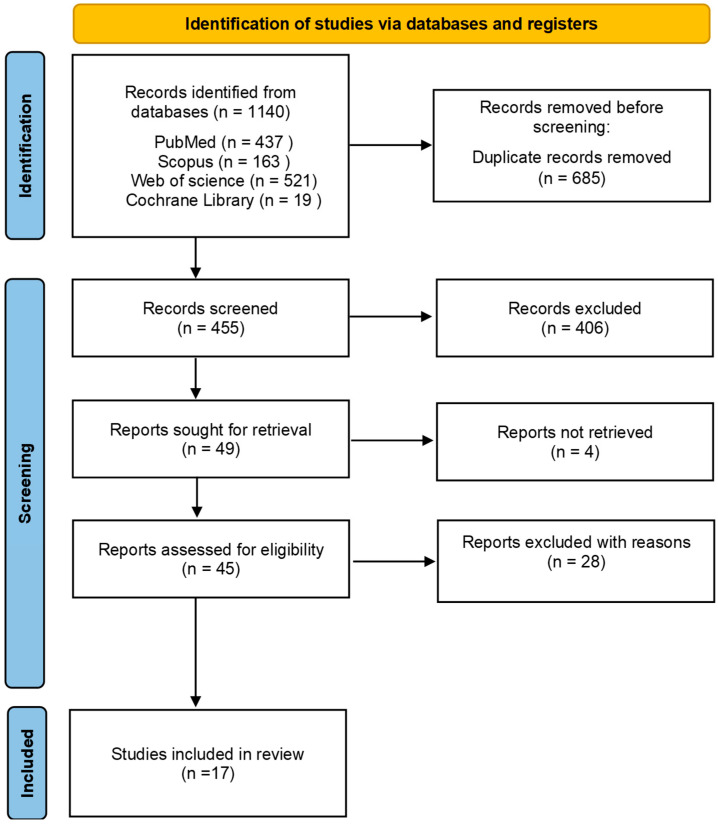
PRISMA flow diagram illustrating the study selection process.

The study selection process is illustrated in the PRISMA flow diagram (Figure 1). A total of 1140 records were initially identified through database searching, including PubMed (*n* = 437), Scopus (*n* = 163), Web of Science (*n* = 521), and the Cochrane Library (*n* = 19). Following the removal of duplicate records (*n* = 685), 455 unique studies remained and were screened based on titles and abstracts. Of these, 406 records were excluded as they did not meet the predefined inclusion criteria.

The full texts of 49 potentially eligible studies were sought; however, 4 reports could not be retrieved. Subsequently, 45 full-text articles were assessed for eligibility, of which 28 were excluded due to reasons such as lack of relevant anatomical data, inappropriate study design, or insufficient reporting. The main reasons for exclusion at the full-text stage included lack of relevant anatomical data, inappropriate study design (e.g., case reports or reviews), and insufficient reporting of splenic artery variations. Ultimately, 17 studies met the inclusion criteria and were considered in the final qualitative synthesis.

### 3.2. Study Characteristics

A total of 17 studies were included in the final analysis, representing a range of methodological approaches, including descriptive cadaveric studies, radiological imaging-based analyses, and retrospective clinical studies with surgical correlation. Sample sizes varied widely, from small cadaveric series (*n* = 26) to large imaging cohorts exceeding 1500 subjects, reflecting significant heterogeneity in study design and scope. The studies were conducted across diverse geographic regions, including Asia, Europe, Africa, the Middle East, and South America.

Cadaveric studies primarily provided direct anatomical insights into terminal branching patterns, origin variability, and segmental distribution of the splenic artery [2,20,21,22,23,24,25]. Radiological studies mainly using computed tomography angiography (CTA) or multidetector CT (MDCT) offered detailed in vivo visualization and morphometric evaluations, including vessel length, diameter, and tortuosity indices [26,27,28,29,30,31,32]. Clinical studies further contributed by correlating anatomical variations with intraoperative findings and surgical outcomes, such as the impact of the splenic artery origin on operative time, blood loss, and the difficulty of lymph node dissection [33,34,35].

Across the selected studies, the most frequently assessed parameters were variations in origin (e.g., from the celiac trunk, aorta, or superior mesenteric artery), course in relation to the pancreas (suprapancreatic, retropancreatic, or intrapancreatic), and terminal branching patterns, with several studies also reporting accessory or replaced vessels and their relevance to pre-surgical planning, embolization safety, and the prevention of iatrogenic injury. A detailed overview of study design, sample size, methodological approaches, anatomical parameters, and key findings is provided in Supplementary Table S1.

**Figure 2:**
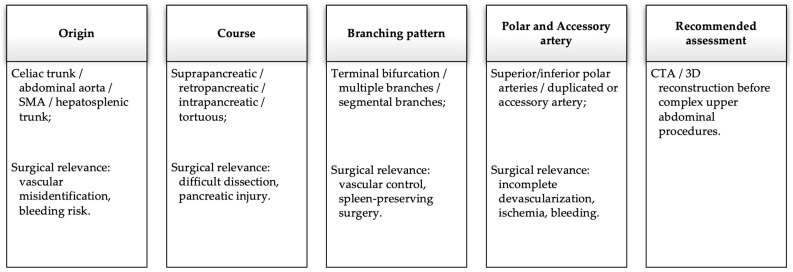
Schematic overview of splenic artery anatomical variations and clinical implications. CTA—Computed Tomography Angiography; SMA—Superior Mesenteric Artery.

To improve readability, the principal anatomical variations in the splenic artery and their clinical implications are summarised schematically in Figure 2.

The figure groups the main variants according to origin, course, branching pattern, and accessory vessels, and links each category to its relevance for pancreatic, gastric, splenic, and endovascular procedures.

The complete study characteristics, risk of bias assessment, and range-based synthesis of the reported anatomical variations are provided in Supplementary Tables S1–S3.

### 3.3. Methodological Quality

The methodological quality and risk of bias of the included studies varied according to study design and methodological approach. Most retrospective imaging and clinical studies demonstrated a consistently low risk of bias, reflecting strong methodological rigour and reliable reporting [26,28,29,31,32,33]. In contrast, descriptive cadaveric studies showed greater variability due to less standardised methodology and inconsistent anatomical reporting [20,23,24,25]. A few studies presented minor concerns in reporting or anatomical detail but did not significantly affect overall quality [27,30]. The full AQUA-based risk of bias assessment is provided in Supplementary Table S2.

### 3.4. Anatomical Variations in the Splenic Artery

#### 3.4.1. Origin of the Splenic Artery

The splenic artery most commonly originates from the celiac trunk, reported in over 90% of cases across both cadaveric and radiological studies, with values ranging from 90.6% in cadaveric series to as high as 98.6% in CTA-based analyses [3,27]. Consistent findings are also observed in large imaging cohorts, where celiac trunk origin has been reported in approximately 93.8% of cases [35]. Less frequent origins from the abdominal aorta occur in a small proportion of cases, typically ranging from 1.4% to 8.1% [27], while origins from the superior mesenteric artery are rare, reported in only 0.13% to 0.7% of cases [10,35]. In addition, variant origins from hepatosplenic or other composite trunks have also been reported, reflecting the complexity of celiac axis branching patterns [10].

#### 3.4.2. Course

A suprapancreatic course along the superior border of the pancreas represents the predominant anatomical pattern, observed in approximately 74–81% of cases [27,31,35]. However, variations including retropancreatic (up to ~16.8%) and intrapancreatic courses are consistently reported [35]. The splenic artery frequently followed a tortuous course, with studies reporting tortuosity in more than half of cases (approximately 54–62%) and looping patterns in up to 86% [32,34]. These variations in arterial course and their relationship to the pancreas are clinically relevant, as deeper, concealed, tortuous, or looping courses may increase the difficulty of vascular exposure, proximal control, lymph node dissection, and endovascular catheter navigation [17].

#### 3.4.3. Branching Pattern and Terminal Distribution

Branching patterns of the splenic artery are highly variable, although terminal bifurcation is the most common pattern, observed in approximately 63–85% of cases [10,27]. The number of terminal branches may range from two to more than six, reflecting considerable heterogeneity in vascular distribution [27]. Cadaveric studies further identify distinct distribution patterns, with the distributed type reported in 84% and the magistral type in 16% of cases [30]. Segmental branching typically ranges from 2 to 5 branches, most commonly three (66.6%), with frequent presence of polar arteries [29,34]. Variations in gastric branches are also notable, with the left gastroepiploic artery arising from the splenic artery in up to 100% of cases in some studies and short gastric arteries commonly originating from superior terminal branches [28,30].

#### 3.4.4. Accessory and Rare Variations

Accessory splenic arteries and rare anomalies were infrequently reported across the included studies. Duplicated splenic arteries were observed in approximately 0.13% of cases, while complete absence of the splenic artery was identified in 0.53 [13]. Additional variations included atypical origins from variant trunks, such as hepatosplenic configurations or direct origin from the abdominal aorta [13,14]. These findings highlight the presence of uncommon but diverse anatomical variants within the splenic arterial system.

### 3.5. Surgical Implications

Anatomical variations in the splenic artery have direct implications for pancreatic, gastric, splenic, and endovascular procedures. Unusual origins from the abdominal aorta or superior mesenteric artery may increase the risk of vascular misidentification and injury [10,33]. Retropancreatic, intrapancreatic, tortuous, or looping courses can complicate arterial exposure, lymph node dissection, distal pancreatectomy, and endovascular access [17]. Branching variability, including early division, segmental branches, polar arteries, and accessory vessels, is particularly relevant for vascular control, spleen-preserving procedures, embolization, and the prevention of ischemic complications [27,29]. These findings support the use of preoperative CTA or 3D vascular reconstruction in selected complex upper abdominal procedures [28,30].

## 4. Discussion

### 4.1. Principal Findings

The findings of this review indicate that the splenic artery exhibits substantial anatomical variability in its origin, course, and branching pattern, with consistent trends observed across cadaveric, radiological, and clinical studies. Despite this variability, a celiac trunk origin and suprapancreatic course remain the predominant anatomical configurations. These findings are in agreement with classical anatomical descriptions, which report the splenic artery as the largest branch of the celiac trunk with a characteristically tortuous course along the superior border of the pancreas [11,31].

A major contribution of the present review is the integration of cadaveric, radiological, and clinically correlated studies into a unified analysis of splenic artery variability. Previous literature has often evaluated these findings separately, particularly focusing on descriptive cadaveric anatomy. By combining anatomical, imaging-based, and surgical evidence, this review highlights the translational relevance of splenic artery variations and emphasizes their practical implications for preoperative planning, minimally invasive surgery, and interventional procedures.

### 4.2. Comparison with Previous Literature

In particular, the systematic review by Manatakis et al. summarised splenic artery variants based mainly on cadaveric evidence, with only limited integration of radiological and clinical data [36].

Although less frequent, variant origins of the splenic artery remain clinically important because they may alter the expected vascular anatomy and complicate surgical or endovascular procedures, notably in minimally invasive approaches. Similar findings have been reported in anatomical literature, where variant origins are associated with complex celiac axis configurations and may complicate vascular ligation and dissection [28]. The identification of such variations preoperatively is therefore critical, predominantly in minimally invasive procedures where visual and tactile feedback is limited.

The relationship of the splenic artery to the pancreas appears to be one of the most clinically relevant anatomical features because concealed or deep arterial courses may increase operative complexity during gastrectomy and distal pancreatectomy. These deeper courses are associated with increased intraoperative complexity, as they require more extensive dissection and carry a higher risk of pancreatic injury [7,35]. Clinical studies included in this review further support this association, demonstrating that concealed or pancreas-covered arteries are linked to longer operative time, increased blood loss, and reduced lymph node retrieval during gastrectomy [17,34]. These findings reinforce the importance of understanding the spatial relationship between the splenic artery and pancreas in surgical strategy [34,35].

Tortuosity of the splenic artery is another consistent finding across studies and represents a relevant technical factor in both open and minimally invasive procedures. During distal pancreatectomy, splenectomy, and spleen-preserving pancreatic surgery, a tortuous or looping artery may complicate vascular exposure, proximal control, and safe dissection along the superior border of the pancreas. Pronounced tortuosity may also increase the risk of inadvertent arterial traction, tearing, or bleeding, particularly when the artery is partially embedded in pancreatic tissue or located in a retropancreatic position. In laparoscopic and robotic procedures, these challenges may be amplified by limited tactile feedback and restricted angles of approach, making preoperative recognition of the arterial course particularly important [32,34,35].

From an endovascular perspective, tortuosity may affect catheter stability, guidewire advancement, selective catheterisation of distal branches, and controlled delivery of embolic material. These technical difficulties are especially relevant during splenic artery embolization, treatment of splenic artery aneurysms or pseudoaneurysms, and preoperative embolization before splenectomy. Severe tortuosity may require the use of microcatheters, shaped catheters, more flexible guidewires, or alternative catheterisation strategies to achieve safe distal access while minimising the risk of arterial spasm, dissection, perforation, or non-target embolization. Therefore, assessment of tortuosity on preoperative CTA or 3D vascular reconstruction should be considered an important component of procedural planning [32,34,35].

Branching variability is particularly important in spleen-preserving procedures because segmental arterial distribution and limited intersegmental anastomoses directly influence splenic perfusion. Previous anatomical studies have emphasised that the splenic artery supplies the spleen in a segmental manner with limited intersegmental anastomosis, supporting the feasibility of partial splenectomy when vascular anatomy is well understood [29].

Variations in the origin and distribution of gastric branches, including the left gastroepiploic and short gastric arteries, further contribute to surgical complexity [32]. These vessels play a critical role in gastric perfusion and are frequently encountered during gastrectomy and lymph node dissection. Consistent findings from cadaveric studies highlight the importance of preserving these branches to prevent ischemic complications, particularly in procedures involving extensive lymphadenectomy [28,30].

### 4.3. Clinical Implications and Preoperative Assessment

The role of preoperative imaging has been strongly emphasized across radiological and clinical studies. Advanced imaging modalities such as CTA and 3D reconstruction enable detailed visualization of vascular anatomy and allow for accurate identification of anatomical variations [30,31,32,33,34,35].

In interventional radiology, knowledge of splenic artery segmentation and branching is essential for safe and effective embolization [30,31,32,33,34,35]. Cadaveric and angiographic studies have identified specific anatomical landmarks and safe zones for embolization, pointing out the need to avoid non-target ischemia and complications such as pancreatitis [26]. These considerations highlight the importance of integrating anatomical knowledge with imaging techniques in both surgical and interventional settings. Overall, the findings of this review confirm that while certain anatomical patterns of the splenic artery are predominant, significant variability exists across all parameters. This variability underscores the necessity of detailed anatomical understanding and individualized preoperative assessment.

From a clinical standpoint, certain anatomical configurations should be classified high-risk during upper abdominal procedures. In particular, retropancreatic or intrapancreatic courses of the splenic artery may significantly increase surgical complexity and the risk of vascular or pancreatic injury. Similarly, early branching patterns and the presence of polar arteries require careful intraoperative identification to avoid incomplete splenic perfusion or ischemic complications.

Several surgical techniques are directly influenced by splenic artery anatomy. In distal pancreatectomy, early identification and control of the splenic artery are essential to reduce blood loss and avoid pancreatic injury, particularly when the artery follows a retropancreatic, intrapancreatic, or markedly tortuous course.

In spleen-preserving distal pancreatectomy, preservation of the splenic artery and vein requires careful dissection along the pancreatic body and tail, whereas Warshaw-type procedures rely on preservation of the short gastric and left gastroepiploic vessels to maintain splenic perfusion. In splenectomy and partial splenectomy, knowledge of terminal branching, segmental arteries, and polar arteries is important for selective vascular control and prevention of residual bleeding or ischemia. During gastrectomy with lymphadenectomy, the splenic artery serves as an anatomical landmark for lymph node dissection along the suprapancreatic border, and variants in its course or branching may increase the risk of vascular injury.

In endovascular procedures, including splenic artery embolization and treatment of aneurysms or pseudoaneurysms, tortuosity, branching angle, and segmental distribution influence catheter selection, distal access, embolization site, and the risk of non-target ischemia.

From a clinical perspective, the most surgically relevant variants identified in this review include retropancreatic or intrapancreatic arterial courses, early terminal branching, and the presence of polar or accessory arteries. Variants involving a concealed or pancreas-covered splenic artery appear particularly critical during gastrectomy and distal pancreatectomy because they may increase intraoperative complexity and complicate vascular dissection. Likewise, early branching patterns and segmental arterial distribution are highly relevant in spleen-preserving procedures, where incomplete identification of accessory or polar vessels may compromise splenic perfusion or increase bleeding risk. These findings support the routine use of detailed preoperative vascular imaging, particularly in complex upper abdominal surgery.

Despite the growing number of studies, the literature remains heterogeneous, with inconsistent classification systems and variability in reporting anatomical parameters. This lack of standardization limits comparability between studies and highlights the need for a unified classification system for splenic artery variations.

Anatomical variations in the splenic artery arise from abnormal persistence or regression of embryonic anastomoses during fetal development. These variations can result in the splenic artery arising from different sources or exhibiting atypical branching patterns [23].

Standardised preoperative reporting of splenic artery anatomy should be encouraged, particularly in radiological assessments, to improve communication between radiologists and surgeons (Box 1).

To improve the translational applicability of the reviewed evidence, a practical preoperative assessment checklist was developed based on the most clinically relevant anatomical variants identified across included studies (Box 1).

Box 1 **Preoperative Assessment Checklist for Splenic Surgery.**

**SUGGESTED PREOPERATIVE IMAGING ASSESSMENT (MDCT Angiography recommended):**

Confirm splenic artery origin;

Document arterial trajectory;

Count and map terminal branches;

Particular attention should be given to: actively search for accessory splenic arteries.

**INTRAOPERATIVE STRATEGY:**

• Anatomic variants identified preoperatively may complicate laparoscopic visualization and require individualized intraoperative decision-making.

• Non-celiac trunk origin → Anticipate altered spatial relationships and vascular anatomy.

• Multiple terminal branches (>5): Consider a selective arterial ligation strategy.

**POSTOPERATIVE VIGILANCE:**

• Unexplained postoperative bleeding → Suspect unidentified accessory arterial supply.

• Residual splenic tissue on follow-up imaging → Evaluate for accessory vessel perfusion.

CTr: Celiac trunk; SMA: Superior mesenteric artery; MDCT: Multidetector computed tomography.

Future research should focus on standardized classification systems for splenic artery variations, the relationship between anatomical variations and surgical outcomes, and the role of advanced imaging techniques and intraoperative technologies. Larger, multicenter studies are also needed to improve generalizability and understand population-based differences.

Overall, integrating anatomical research with clinical application can be essential to optimize preoperative planning and outcomes in upper abdominal procedures.

### 4.4. Limitations

This review has several limitations. Restriction to English-language publications may have introduced language bias and potentially excluded relevant studies published in other languages. In addition, the review was not prospectively registered in a database such as PROSPERO, which may reduce methodological transparency despite the use of predefined review methods. The included studies were methodologically heterogeneous, comprising cadaveric, radiological, and clinical investigations that are not always directly comparable.

Furthermore, some included studies were not primarily designed to evaluate splenic artery variations, which may have influenced the consistency and depth of anatomical reporting. Variability in reporting of anatomical findings, together with the absence of standardized definitions for arterial course, tortuosity, accessory arteries, and branching patterns, further limited comparability across studies and complicated synthesis of the available evidence. Surgical and clinical outcomes were inconsistently reported, limiting the ability to draw robust conclusions regarding operative risk and clinical impact. Finally, the heterogeneity of included studies and the qualitative nature of the available evidence precluded formal quantitative meta-analysis.

## 5. Conclusions

The splenic artery exhibits substantial anatomical variability and should not be assumed to follow a predictable anatomical pattern, but rather as a highly variable vessel with direct consequences for upper abdominal surgery and endovascular intervention. This systematic review shows that variations in origin, course, tortuosity, terminal branching, and accessory or polar arteries may influence surgical exposure, vascular control, lymph node dissection, splenic perfusion, and the risk of intraoperative bleeding or ischemic complications.

The key clinical message is that individualized assessment of vascular anatomy is essential. Retropancreatic or intrapancreatic arterial courses, marked tortuosity, early terminal branching, and polar or accessory arteries should be actively identified before pancreatic, gastric, splenic, and endovascular procedures. Preoperative CTA, preferably combined with three-dimensional vascular reconstruction when available, can support safer operative planning and more individualized surgical strategies.

Overall, integrating anatomical knowledge with modern imaging and surgical decision-making may contribute to reducing vascular complications and improve procedural safety in upper abdominal surgery.

## Figures and Tables

**Table 1:** Eligibility criteria according to a PECO-style framework.

Component	Description
Population	Human subjects and cadaveric specimens
Exposure	Anatomical variations in the splenic artery
Outcomes	Variations in origin, course, branching pattern, accessory arteries, and reported surgical implications
Study types	Cadaveric studies, radiological studies, retrospective or prospective observational clinical studies
Exclusion criteria	Case reports, reviews, editorials, conference abstracts, animal studies, and non-English publications

## Data Availability

Not applicable. The original contributions presented in this study are included in the article. Further inquiries can be directed to the corresponding authors.

## Supplementary Materials

The following supporting information can be downloaded at: https://www.mdpi.com/article/10.3390/life16071077/s1, Table S1: Summary of study characteristics and key anatomical findings of the splenic artery across included studies. Table S2: Risk of bias assessment of included studies using the AQUA tool. Table S3: Range-based synthesis of reported anatomical variations of the splenic artery across included studies.

## Abbreviations

The following abbreviations are used in this manuscript:

CT	Computed Tomography
CTr	Celiac Trunk
AA	Abdominal Aorta
SMA	Superior Mesenteric Artery
CMT	Celiaco-mesenteric trunk
HMT	Hepato-mesenteric trunk
ROS	Retrospective observational study
CR	Case report
CSS	Cross-Sectional study
LGEA	Left gastroepiploic artery
MDCT	Multidetector computed tomography
Angio-CT	Angiography Computed Tomography
Angio-MRI	Magnetic Resonance Angiography
