# Supplementary material for: Anatomical Variations of the Splenic Artery: Clinical and Surgical Implications—A Systematic Review
Source: Life (Basel). 2026 Jun 27;16(7):1077. doi: 10.3390/life16071077 (PMC13413164; doi:10.3390/life16071077)
Supplement: Supplementary file 1 [file life-16-01077-s001.zip › life-4311341-supplementary.pdf]

Author (Year)	Study Design	Sample Size	Methodological Approach	Origin	Course	Branching Pattern	Clinical Relevance
Ekingen et al. (2020) [26]	Retrospective observational study	750	Radiological (MDCT angiography)	CT 79.47%; aorta 1.2%; SMA 0.13%; hepatosplenic trunk 4%	NR	23 branching variants; double SA 0.13%; absent SA 0.53%	Relevant for vascular and surgical planning
Sheta (2024) [27]	Retrospective observational study	389	Radiological (angiography)	CT predominant; aorta 0.51%	NR	LGA from SA 0.51%	Relevant for surgical planning
Saldarriaga et al. (2023) [25]	Descriptive cadaveric study	26	Cadaveric	Aorta 7.7%	Tortuous 54%; straight 31%	Terminal bifurcation predominant	Associated with technical complexity during surgery
Zhu et al. (2018) [33]	Retrospective observational study	169	Radiological (CT-based 3D reconstruction)	NR	Superficial 36.7%; middle 49.1%; concealed 14.2%	NR	Associated with greater surgical difficulty
Naga Jyothi et al. (2015) [23]	Descriptive cadaveric study	50	Cadaveric	NR	NR	Distributed type 84%; magistral 16%; accessory LGA 2%	Relevant for spleen-preserving surgery
Brinkman et al. (2021) [28]	Retrospective observational study	80	Radiological (contrast-enhanced CT with 3D reconstruction)	Ct 100%	Looping morphology present in 86%	Proximal polar branches 59%	Important for surgical anatomical assessment
Bilek et al. (2024) [29]	Retrospective observational study	274	CTA with multiplanar and 3D reconstruction	CT 93.8%; aorta 5.5%; SMA 0.7%	Superior 81%; retropancreatic 16.8%	Terminal bifurcation predominant; Y-type pattern 65%	Relevant for pancreatic and splenic surgery
Fataftah et al. (2020) [30]	Retrospective observational study	219	Radiological (CT scan analysis)	NR	Predominantly tortuous course	NR	Associated with increased operative difficulty
Gumede et al. (2025) [24]	Descriptive observational study	32	Dissection with direct morphological observation	CT 93.8%; aorta 6.3%	Suprapancreatic 100%	NR	Associated with increased operative complexity
Ndoye et al. (2008) [21]	Descriptive observational study	79	Dissection and arterial opacification (barium injection, radiography)	NR	NR	LGEA from SA 100%; Type II most common	Relevant for spleen-preserving procedures
Wilk et al. (2025) [31]	Retrospective observational study	74	CTA with 3D reconstruction	CT 98.6%; aorta 1.4%	Tortuous 62.2%; retropancreatic 50%	Common trunk pattern 69.9%	Relevant for surgical anatomical assessment
Fukada et al. (2025) [34]	Retrospective observational study	123	Radiological analysis with clinical correlation	NR	Pancreas-covered SA (Type 2)	NR	Associated with longer operative time
Sindel et al. (2001) [20]	Descriptive cadaveric study	22	Cadaveric	NR	NR	SA divided into 4 segments	Important for safe embolization
Chaware et al. (2012) [22]	Descriptive cadaveric study	111	Cadaveric	NR	NR	2–5 segmental branches; polar branches frequent	NR
Chen et al. (2015) [32]	Retrospective observational study	1551	Radiological (MDCT + 3D reconstruction) + intraoperative surgical analysis	Low rate of anatomical variation (7%)	Close relationship with the suprapancreatic border	NR	Proximal SA approach associated with reduced blood loss
Pandey et al. (2004) [2]	Descriptive cadaveric study	320	Cadaveric	CT 90.6%; aorta 8.1%	Suprapancreatic 74.1%	2–6 terminal branches; bifurcation most common	Relevant for splenic and pancreatic surgery
Ikeda et al. (2020) [35]	Retrospective observational study	32	Radiological (3D-CT simulation) + intraoperative video analysis	NR	Relation to surrounding structures assessed by 3D-CT	NR	3D-CT improved surgical efficiency

Supplementary Table S1. Summary of study characteristics and key anatomical findings of the splenic artery across included studies.

SA, splenic artery; CT, celiac trunk; SMA, superior mesenteric artery; LGA, left gastric artery; LGEA, left gastroepiploic artery; SPA, superior polar artery; IPA, inferior polar artery; CTA, computed tomography angiography; CT, computed tomography; MDCT, multidetector computed tomography; MPR, multiplanar reconstruction; MIP, maximum intensity projection; VR, volume rendering; LN, lymph node; NR, Not reported. Y-type refers to a terminal branching pattern of the splenic artery according to the IPALGEA classification proposed by Bilek et al.

Supplementary Table S2. Risk of bias assessment of included studies using the AQUA tool.

Reference	Study Design	Domain 1	Domain 2	Domain 3	Domain 4	Domain 5
Ekingen et al. (2020) [26]	Retrospective observational study	Low	Low	Low	Low	Low
Sheta (2024) [27]	Retrospective observational study	Low	Low	Low	Low	Unclear
Saldarriaga et al. (2023) [25]	Descriptive observational study	Low	High	High	Low	Low
Zhu et al. (2018) [33]	Retrospective observational study	Low	Low	Low	Low	Low
Naga Jyothi et al. (2015) [23]	Descriptive observational study	Low	High	High	Low	Low
Brinkman et al. (2021) [28]	Retrospective observational study	Low	Low	Low	Low	Low
Bilek et al. (2024) [29]	Retrospective observational study	Low	Low	Low	Low	Low
Fataftah et al. (2020) [30]	Retrospective observational study	Low	Low	Low	High	Low
Gumede et al. (2025) [24]	Descriptive observational study	Low	High	High	Low	Low
Ndoye et al. (2008) [21]	Descriptive observational study	Low	Low	Low	Low	Low
Wilk et al. (2025) [31]	Retrospective observational study	Low	Low	Low	Low	Low
Fukada et al. (2025) [34]	Retrospective observational study	Low	Low	Low	High	Low
Sindel et al. (2001) [20]	Observational study	Low	High	Low	High	Low
Chaware et al. (2012) [22]	Descriptive observational study	Low	Low	High	Low	Low
Chen et al. (2015) [32]	Retrospective observational study	Low	Low	Low	High	Low
Pandey et al. (2004) [2]	Descriptive observational study	Low	Low	Low	Low	Low
Ikeda et al. (2020) [35]	Retrospective observational study	Low	Low	Low	High	Low

Anatomical Quality Assurance (AQUA) tool across five domains: Domain 1 (objectives and subject characteristics), Domain 2 (study design), Domain 3 (methodological characterization), Domain 4 (descriptive anatomy), and Domain 5 (reporting of results). Studies were categorized as low, high, or unclear risk of bias based on predefined AQUA criteria. Study designs were classified as observational retrospective (clinical or imaging-based) or descriptive (cadaveric/anatomical) according to methodological approach. "Low," "High," and "Unclear" refer to the estimated risk of bias within each AQUA domain and not to the overall quality of the study. "Low" indicates low risk of bias, "High" indicates high risk of bias, and "Unclear" indicates insufficient methodological information for adequate assessment.

Suplemmentary Table S3. Range-based synthesis of reported anatomical variations of the splenic artery across included studies

Anatomical Domain	Most Common Finding	Reported Range Across Studies	Main Study Types
Origin from celiac trunk	Predominant arterial origin	79.4–100%	Cadaveric + radiological
Origin from abdominal aorta	Rare anatomical variant	0.5–8.1%	Cadaveric + radiological
Origin from SMA	Very rare variant	0.1–0.7%	Radiological
Hepatosplenic or composite trunk origin	Uncommon variant origin	Up to 4%	Radiological
Suprapancreatic course	Most common arterial course	74–100%	Cadaveric + radiological
Retropancreatic course	Less frequent variant	16.8–50%	Radiological
Intrapancreatic/concealed course	Rare but clinically relevant variant	Up to 14.2%	Radiological + clinical
Tortuous/looping morphology	Frequent morphological feature	54–86%	Cadaveric + radiological
Terminal bifurcation	Most common branching pattern	63–84.7%	Cadaveric + radiological
Segmental branching	Usually 2–5 segmental branches	Most commonly 3 branches	Cadaveric
Distributed branching pattern	Predominant branching type	84%	Cadaveric
Magistral branching pattern	Less common branching type	16%	Cadaveric
Polar arteries	Frequently identified	55–59%	Cadaveric + radiological
Left gastroepiploic artery from SA	Common anatomical finding	84–100%	Cadaveric
Accessory splenic artery	Rare variation	0.1–0.5%	Radiological
Absent splenic artery	Extremely rare variation	0.5%	Radiological
Pancreas-covered splenic artery	Associated with increased operative complexity	Up to 14.2%	Clinical + radiological
3D-CT vascular mapping	Associated with improved operative planning	Reported qualitative benefit	Clinical + radiological
